# Indolent Ulcer Successfully Treated by Compression

**Published:** 1869-02

**Authors:** J. F. Grimes

**Affiliations:** Wapello, Iowa


### INDOLENT ULCER SUCCESSFULLY TREATED BY COMPRESSION.

BY J. F. GRIMES, M. D., WAPELLO, IOWA.

Mrs. Hartman, a German lady, aged 49 years, applied to me August 30th, 1867. Upon examination, I found an indolent ulcer of left leg, extending from internal malleolus to junction of middle with lower third of tibia, and from crest of tibia one and a half inches on internal aspect. She had been troubled with this ulcer for more than fifteen years; had been treated by various physicians with but temporary relief. It had healed several times, but ulceration and renewal of the discharge as often followed, with increased deposit and hardening of the surrounding structures. The treatment adopted was invariably stimulating applications, without reference to pain induced, or irritability of system existing. The changes naturally following such treatment can be readily understood by the intelligent physician. The patient was apparently in good health, except slight anorexia, which I attributed more to a malarial cause than otherwise, and gave her the following prescription: *R.* Fluid ext. cinchona; syrup simplex aa. ounces jss; spirits Frumenti, one ounce, M. S.; tablespoonful three or four times a day, which had the desired effect. Then taking the common adhesive plaster, I cut it in strips about 1¼ inch in width and long enough to reach two-thirds around the leg in an oblique direction, and commenced its application by placing the first strip below the ulcer and the parts indurated, applying them firmly and in a spiral direction each way, alternately overlaping each other in a kind of plat until the ulcer and indurated parts were entirely covered. I then made a small aperture at the lower margin for the escape of pus. In twenty-four hours the strips were removed and fresh ones applied. This treatment was continued about three weeks, at which time I found a marked improvement in the condition of the ulcer and its surroundings. Absorption was rapidly going on in the elevated edges; discharge had materially lessened, and its quality improved, granulation presenting of a healthy character. In fact, I had an indolent ulcer assuming the character of a healthy sore. Knowing the disposition of such ulcers to assume the weak condition, I continued the dressings for several weeks, removing the plasters at intervals of two, three or four days, until cicatrization was perfected and recovery complete.

